# Novel Insights into the Predictors of Obstructive Sleep Apnea Syndrome in Patients with Chronic Coronary Syndrome: Development of a Predicting Model

**DOI:** 10.1155/2022/5497134

**Published:** 2022-06-27

**Authors:** Yanan Xu, Zongwei Ye, Benfang Wang, Long Tang, Jun Sun, Xuedong Chen, Yi Yang, Jun Wang

**Affiliations:** ^1^Pulmonary and Critical Care Medicine, People's Hospital of Xuancheng City, Anhui, China; ^2^Suzhou Ninth Hospital Affiliated to Soochow University, Jiang Su, China; ^3^Department of Cardiology, The First Affiliated Hospital of Bengbu Medical College, Bengbu, Anhui, China; ^4^Department of Cardiology, People's Hospital of Xuancheng City, Anhui, China; ^5^Xinjiang Medical University, Urumqi 830011, China; ^6^Department of Cardiology Fourth Ward, The Xinjiang Medical University Affiliated Hospital of Traditional Chinese Medicine, Urumqi 830011, China

## Abstract

**Background:**

Obstructive sleep apnea syndrome (OSAS) is common in patients with chronic coronary syndrome (CCS); however, a predictive model of OSAS in patients with CCS remains rarely reported. The study aimed to construct a novel nomogram scoring system to predict OSAS comorbidity in patients with CCS.

**Methods:**

Consecutive CCS patients scheduled for sleep monitoring at our hospital from January 2019 to September 2020 were enrolled in the current study. Coronary CT angiography or coronary angiography was used for the diagnosis of CCS, and clinical characteristics of the patients were collected. Significant predictors for OSAS in patients with moderate/severe CCS were estimated via logistic regression analysis, and a clinical nomogram was constructed. A calibration plot, examining discrimination (Harrell's concordance index) and decision curve analysis (DCA), was applied to validate the nomogram's predictive performance. Internal validity of the predictive model was assessed using bootstrapping (1000 replications).

**Results:**

The nomograms were constructed based on available clinical variables from 527 patients which were significantly associated with moderate/severe OSAS in patients with CCS, including body mass index, impaired glucose tolerance, hypertension, diabetes mellitus, nonalcoholic fatty liver disease, and routine laboratory indices such as neutrophil to lymphocyte ratio, platelet-to-lymphocyte ratio, high-density lipoprotein cholesterol, and low-density lipoprotein cholesterol. The C-index (0.793) and AUC (0.771, 95% CI: 0.731–0.811) demonstrated a favorable discriminative ability of the nomogram. Moreover, calibration plots revealed consistency between moderate/severe OSAS predicted by the nomogram and validated by the results of sleep monitoring. Clinically, DCA showed that the nomogram had good discriminative ability to predict moderate/severe OSAS in patients with CCS.

**Conclusions:**

The risk nomogram constructed via the routinely available clinical variables in patients with CCS showed satisfying discriminative ability to predict comorbid moderate/severe OSAS, which may be useful for identification of high-risk patients with OSAS in patients with CCS.

## 1. Introduction

Coronary artery disease (CAD) remains one of the most important causes of mortality worldwide [1, 2]. Although effective revascularization and risk-factor targeted therapy have substantially improved the survival of patients with CAD, patients with CAD continue to suffer from excessive morbidity and mortality [3, 4]. Obstructive sleep apnea syndrome (OSAS) is common in the general population, with reported prevalence of 20%–30% [5, 6]. Pathologically, OSAS is characterized by intermittent hypoxia and consequent activated inflammation, oxidative stress, and sympathetic system, all of which are involved in the pathogenesis of CAD [7, 8]. Previous reports have shown that OSAS confers an increased risk of CAD, particularly in the high-risk population, such as those with hyperlipidemia, diabetes, and hypertension [9–11]. Indeed, the prevalence of OSAS in patients with CAD is estimated 40–60% [12, 13]. Moreover, OSAS is significantly associated with the coronary plaque burden [14]. Moderate to severe OSAS is consistently shown to be a risk factor of recurrent cardiovascular events in patients with CAD despite of optimal medical treatment [15, 16]. Therefore, a comprehensive understanding of the clinical factors associated with moderate to severe OSAS in patients with CAD may provide additional important prognostic indicators for these patients. Notably, confirmation of OSAS relies on sleep monitoring studies, which is time consuming, associated with high expense and inconvenience in real-world clinical practice. Currently, investigation into a novel risk assessment tool is warranted. Although STOP-BANG and Berlin questionnaire have been proposed as the effective method for screening for detecting OSAS patients, the low specificity and moderate accuracy of the above screening questionnaire may limit their use for OSAS diagnosis [17]. Thus, the desired more effective and novel tool needs to feature a rapid readout and be easily learned and operated, particularly for CAD patients with moderate to severe OSAS [17–19].

A substantial number of patients with CAD also have chronic coronary syndrome (CCS), which is often overlooked in clinical practice as the prognosis of CCS is comparable with that of acute coronary syndrome [20]. While understanding the risk factors for moderate/severe OSAS in patients with CCS is of clinical significance, relevant studies have been rarely reported. Therefore, in this study, we aimed to identify the possible predictors for OSAS comorbidity in patients with CCS and to construct a clinical nomogram for identification of CCS patients at high risk for OSAS.

## 2. Methods

### 2.1. Patient Population

Patients with a first-time diagnosis of CCS via coronary angiography or coronary CT angiography (CTA) and underwent a successful sleep monitoring study at the Xinjiang Medical University Affiliated Hospital of Traditional Chinese Medicine from January 2019 to September 2020 (*n* = 527) were enrolled in this study. The diagnosis of CCS was in accordance with international guidelines [20]. Patients with the following conditions were excluded: acute coronary syndrome (ACS), chronic or acute cardiac failure, implantation of pacemaker, previous paroxysmal or atrial fibrillation, acute- and chronic-phase inflammatory responses and infection, malignant tumor, with previously diagnosed OSAS, or patients without the results of a sleep monitoring study. The study was approved by the Ethics Committee of Xinjiang Medical University Affiliated Hospital of Traditional Chinese Medicine (No. 2022XE0103-1). As the data is anonymous, informed consent was not required.

### 2.2. Sleep Monitoring Study

For all participants, polysomnography (Grael; Compumedics, Melbourne, VIC, Australia) was performed, and parameters of airflow, thoraco-abdominal movements, pulse oximetry, and snore episodes were recorded. Sleep states and arousal were scored on the basis of standard criteria [21, 22]. Apnea was diagnosed when there was a ≥90% decrease in airflow from the pre-event baseline lasting for ≥10 s. Hypopnea was defined as a ≥30% decrease in airflow from the pre-event baseline lasting for ≥10 s accompanied by either a ≥4% oxygen desaturation or a reduction in airflow of ≥50%, lasting for ≥10 s, accompanied with a 3% oxygen desaturation. Apnea-hypopnea index (AHI) was calculated based on the number of apnea and hypopnea events per hour of sleep. Patients with AHI ≥5 events/h were diagnosed as OSAS and classified as mild (5 ≤ AHI ≤15 events/h), moderate (15 < AHI ≤30 events/h), and severe OSAS (AHI >30 events/h).

### 2.3. Biochemical Tests

Prior to coronary angiography or coronary CTA, all patients underwent routine blood biochemical test to obtain the following parameters: inflammation biomarkers (fibrinogen, white blood cell count, high-sensitivity C-reactive protein (hs-CRP), neutrophil to lymphocyte ratio (NLR), and platelet-to-lymphocyte ratio (PLR)) and the N-terminal pro-brain natriuretic peptide (NT-proBNP). Blood was collected in the early morning after fasting to detect lipid profile (high-density lipoprotein cholesterol (HDL-C), low-density lipoprotein cholesterol (LDL-C), very low-density lipoprotein cholesterol (VLDL-C), total cholesterol (TC), triglyceride (TG), apolipoprotein AI (Apo AI), apolipoprotein B (Apo B), and lipoprotein a).

### 2.4. Holter Monitoring and Echocardiography

A 24-hour Holter monitoring was also performed for each of the included patients to evaluate the heart rate variability (HRV) via the time domain parameters as previously described [23–25]. R peak detection was used to identify normal sinus RR intervals, and then, the standard deviation of all normal sinus RR intervals (SDNN), root means square successive difference (RMSSD), and the standard deviation average of NN intervals (SDANN) were calculated. PNN50 represents the percentage of the number of times that the difference between adjacent normal RR intervals is >50 ms over the total number of NN intervals. The echocardiography was also performed to obtain left ventricular ejection fractions (LVEF) and left ventricular end-diastolic diameter (LVEDD) based on the previously described procedures [26, 27].

### 2.5. Statistical Analysis

The SPSS23 software and R software (version 3.6.1; R Foundation for Statistical Computing, Vienna, Austria) were used for statistical analysis. The calculation of sample size was based on the formula: *n* = *P* × (100 − *P*) × *z*^2^/*d*^2^. We adopted a conventional level of confidence of 95%, with *Z* = 1.96 (considering 95% of confidence interval) and ideal precision of 10% (*d* = 10). Continuous variables with normal distribution were presented as means and standard deviations (SDs), while continuous variables with a skewed distribution were summarized as medians and interquartile ranges (IQRs). The *T*-test was used for continuous variables, and for continuous variables, the nonparametric Mann–Whitney *U* test or the Kruskal-Wallis analysis was employed for analyzing nonnormal distribution. The categorical variables were analyzed and compared using the Chi-square (*χ*^2^) test. A univariate analysis was conducted to identify factors related to moderate to severe OSAS as evidenced by AHI in patients with CCS. *P* < 0.05 suggests statistical significance. By analyzing the statistical significance levels of the features and introducing all selected features, CCS patients at high risk of moderate/severe OSAS could be predicted using the statistically significant predictors. Based on the screening features, the predictive nomograms were constructed based on statistical significance. We built the nomogram using “*rms*” package in R software. Receiver characteristic curve (ROC) was constructed, and the area under the ROC curve was employed to reflective the discriminative efficacy of the risk nomogram for moderate/severe OSAS [28]. Calibration curves were plotted and calculated applying the “*rms*” package, which were presented to evaluate the calibration of the moderate/severe OSAS from patients with CCS risk nomogram. Furthermore, we checked the accuracy of our model by performing bootstrap validation by repeating simple random sampling for 1000 repetitions. With the “*nricens*” package, decision curve analysis (DCA) was employed to evaluate the clinical practicability of nomograms according to the net benefit under different threshold probabilities in patients with CCS [29].

## 3. Results

### 3.1. Patient Characteristics

Overall, 527 patients with CCS were included in the study. The average age of the patients was 56.8 years, and 70.9% of the patients were male. Results of sleep monitoring study showed that 55.2% of the patients had moderate to severe OSAS. Baseline characteristics and the parameters from the sleep monitoring study in CCS patients with no or mild OSAS and moderate to severe OSAS are shown in Tables 1 and 2. Briefly, patients with moderate to severe OSAS were more likely to have hypertension (*P* < 0.001), diabetes mellitus (*P* = 0.003), impaired glucose tolerance (*P* < 0.001), nonalcoholic fatty liver disease (NAFLD) (*P* < 0.001), and higher uric acid (*P* = 0.009), glycated hemoglobin (HbA1c) (*P* = 0.004), LDL-C (*P* < 0.001), TC (*P* = 0.013), NLR (*P* = 0.002), PLR (*P* = 0.011), BMI (*P* < 0.001), neck circumference (*P* < 0.001), and average heart rate (*P* = 0.004), but lower SDANN (*P* = 0.035) and HDL-C (*P* = 0.035). In addition, CCS patients with moderate to severe OSAS also had higher obstructive respiratory disturbance index (*P* < 0.001), time ratio of Oxygen saturation (SpO2) <85% (*P* < 0.001), average blood oxygen saturation (*P* < 0.001), lowest blood oxygen saturation (*P* < 0.001), lowest heart rate during sleep (*P* < 0.001), and mean heart rate (*P* < 0.001) compared with those with no or mild OSAS (Table 2).

### 3.2. Potential Predictors for Moderate to Severe OSAS in Patients with CCS

Multivariable stepwise logistic regression analysis showed that hypertension (OR: 1.865, 95% CI: 1.126–3.089, *P* = 0.015), diabetes mellitus (OR: 2.052, 95% CI: 1.321–3.187, *P* = 0.001), impaired glucose tolerance (OR: 4.049, 95% CI: 2.108–7.774, *P* < 0.001), NAFLD (OR: 1.715, 95% CI: 1.143–2.573, *P* = 0.009), HDL-C (OR: 0.369, 95% CI: 0.170–0.804, *P* = 0.012), LDL-C (OR: 2.224, 95% CI: 1.673–2.957, *P* < 0.001), NLR (OR: 1.279, 95% CI: 1.063–1.538, *P* = 0.009), PLR (OR: 1.006, 95% CI: 1.002–1.009, *P* = 0.002), and BMI (OR: 1.095, 95% CI:1.041–1.152, *P* < 0.001) were independent predictors of moderate/severe OSAS in patients with CCS (Table 3).

### 3.3. Construction of the Nomogram

**Figure 1:**
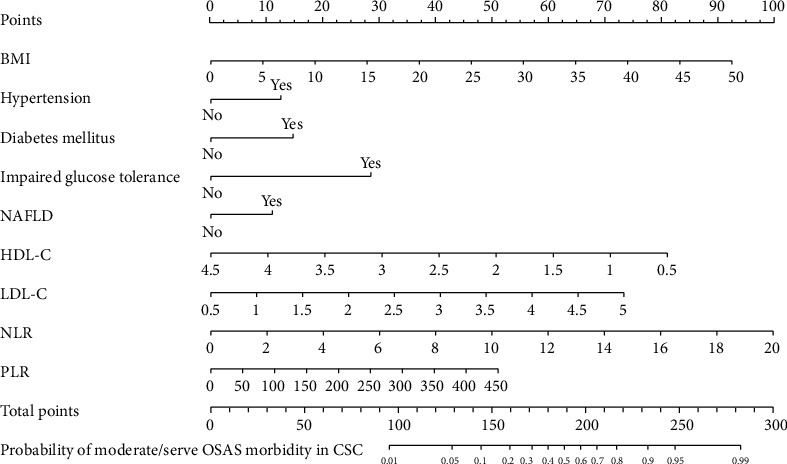
Nomogram and included variables for identification of patients with CCS who are at risk for moderate/severe OSAS. The points according to the presence/absence of the categorized variables and the values of the continuous variables could be obtained according to the value of the horizontal axes. A score based on the added value of all the variables could be then generated, which corresponds to the probabilities of OSAS in individual patient with CCS.

Multivariate logistic regression was employed to screen predictive variables based on those presented in Table 2 and establish the predictive model. A nomogram has been derived from these nine clinic routine variables, including hypertension, diabetes mellitus, impaired glucose tolerance, NAFLD, HDL-C, LDL-C, NLR, PLR, and BMI, as possible predictors (Figure 1). The predictive model was presented as a nomogram, which was applied to quantitatively predict the risk of moderate to severe OSAS in patients with CCS (Figure 1). Total nomogram-related score was calculated for each patient by summing up scores corresponding to the status of variables in the nomogram. All of the evaluated patients with CCS in the current study had total risk points ranging from 0 to 300.

### 3.4. The Performance of the Nomogram

**Figure 2:**
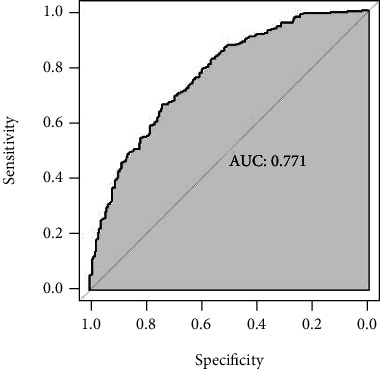
Receiver operating characteristic (ROC) curve analysis to validate the predictive efficacy of the nomogram for patients with moderate/severe OSAS in CCS patients; the true positive rate and false positive rate for the predictive efficacies of the nomogram are shown in the *y*-axis and *x*-axis, separately.

**Figure 3:**
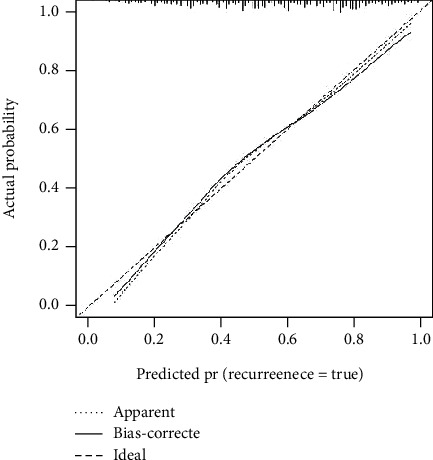
Calibration curves for the predictive efficacy of the risk nomogram for moderate to severe OSAS in patients with CCS. The *y*-axis represents the actual probability of patients with moderate to severe OSAS as validated by the sleep monitoring study, and the *x*-axis represents the predicted risk of moderate to severe OSAS by the risk nomogram. The diagonal dotted line represents a perfect prediction by an ideal model, while the solid line represents the performance of the risk nomogram. A closer fit of the solid line to the diagonal dotted line represents a better prediction.

**Figure 4:**
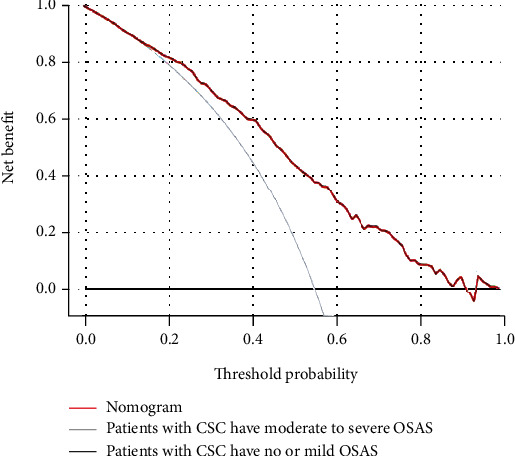
Decision curve analysis (DCA) for the prediction model. The red solid line represents the probability derived from the prediction model, while the gray line presents proportions of patients with moderate to severe OSAS as evidence by the results of the sleep monitoring study. The solid horizontal line indicates the proportions of patients with CCS have no or mild OSAS. The graph depicts the expected net benefit per patient relative to the nomogram prediction of patients with CCS who have moderate to severe OSAS risk. The model curve that is extended indicates the net benefit increases.

We applied ROC curve analysis to assess the discriminatory capacity of the model to detect CCS patients at risk for moderate to severe OSAS (Figure 2). The area under curve (AUC) was 0.771 for detecting CCS patients with moderate to severe OSAS, which indicates that this nomogram is effective to identify CCS patients at risk for moderate to severe OSAS (*P* < 0.001; 95% CI: 0.731–0.811, SE = 0.020, C − index = 0.793). Furthermore, validation of the prediction model was assessed by bootstrap analysis with 1000 replications to estimate the accuracy of the nomogram. The calibration plots were constructed to evaluate the consistency between CCS patients at risk for moderate to severe OSAS who were detected by the nomogram or validated by the results of the sleep monitoring study (Figure 3). The average error rate of model prediction was 0.01. Nomogram prediction and observed observations were well correlated based on calibration curves. Following an evaluation of the accuracy, DCA was also performed which also confirmed the predictive efficacy of the model (Figure 4). The DCA indicated that the threshold probability of the prediction model is 1–87%.

## 4. Discussion

In the current study, by including a cohort of CCS patients who were also subject to a sleep monitoring study, we found multiple clinical characteristics (hypertension, diabetes mellitus, impaired glucose tolerance, NAFLD, and BMI) and biochemical parameters (HDL-C, LDL-C, NLR, and PLR) that were potential predictors for moderate to severe OSAS. Subsequently, a nomogram was constructed and validated to be effective for the prediction of the possible risk of moderate to severe OSAS in patients with CCS. Although validation in future studies is needed, this risk nomogram constructed via routinely available clinical variables in patients with CCS showed satisfying discriminative ability to predict moderate to severe OSAS comorbidity, which may be useful for identification of high-risk patients with moderate to severe OSAS in patients with CCS.

Notably, OSAS and CAD share numerous common risk factors and comorbid conditions such as male gender, advanced age, metabolic syndrome, hyperlipidemia, hypertension, and obesity [30, 31]. In addition, severe and moderate OSAS has been related to poor CAD prognosis, deteriorated sleep-related symptoms, and impaired sleep-related quality of life [30, 31]. In accordance with previous studies, our findings also showed that CCS patients with moderate to severe OSAS were more vulnerable to diabetes mellitus, obesity, hyperlipidemia, and more severe inflammatory response [30–34]. Although OSAS is a well-documented risk for adverse cardiovascular outcomes [6, 13, 30–34], few studies have evaluated the polysomnographic feature of hospitalized CCS patients in previous studies. Moreover, a causative relationship between OSAS and CAD remains to be evaluated because these two diseases share many comorbidities. In addition, few studies have illustrated clinically significant benefits of OSAS treatments on OSA-related CAD outcomes [15, 16]. Furthermore, as the premier method of diagnosing and quantifying OSAS, polysomnography, the “gold standard” for the diagnosis of OSAS, is time-consuming and expensive, which restricts its application in extensive screening. Thus, a simple and cost-effective screening method for the risk of OSAS in patients with CCS is still needed [35]. Additionally, the physiological effects of OASA on cardiovascular structure and function may involve multiple mechanisms, such as intermittent hypoxia, sleep fragmentation, and fluctuation of intrathoracic pressure, which in turn lead to the activation of inflammation, oxidative stress, and sympathetic system. All of these components are important for the pathogenesis of CAD [34].

Moreover, it has been accepted that multiple clinical parameters, such as demographic characteristics, vital signs, laboratory data, and radiological data, are important for the management of patients with CAD, which are usually routinely collected at admission. A previous study indicated that a predictive nomogram based on clinical routine variables is important for early diagnosis and facilitates the development of new prevention and treatment strategies for CAD [36]. Further, a prospective cohort study has confirmed that incorporating available clinical variables into the existing predictive score is important for prognostic estimation after myocardial infarction [37]. While there have been a variety of approaches to create a more effective method and identification of new risk factors to discriminate at high risk for CAD [38–42], no specific prediction model has been developed to identify CCS patients who are at risk for moderate to severe OSAS. Besides, previous studies have demonstrated that Berlin and STOP-BANG questionnaire provides convenient and reliable score tools for OSAS risk assessment, but lack of reliable biomarkers to improve prediction accuracy [17–19]. Additionally, CSC and OSAS shared common risk factors and pathophysiological mechanisms, which may also lead to the common clinical symptoms [7–13]. Thus, we constructed on a risk nomogram integrated approach incorporating the clinical characteristics and biochemical parameters to identify CCS patients with moderate to severe OSAS. In our study, we established a novel scoring system that is feasible and easy to for the identification of CCS patients at risk for moderate to severe OSAS. Potential predictors were screened according to previously described methods [30] and validated in a logistic multiple regression analysis. The C-index (0.793) and AUC (0.771, 95% CI: 0.731–0.811) demonstrated favorable discriminative ability of the nomogram. Moreover, calibration plots revealed satisfying consistency between OSAS predicted by the nomogram and validated by the results of sleep monitoring. Our study supports incorporating these clinical variables to individualized risk assessment and risk stratification in CCS patients with moderate to severe OSAS, which may be helpful for optimal the clinical management of these patients.

### 4.1. Study Limitations

First, the sample size for this retrospective observational study is small. In light of this, these encouraging findings need to be validated in subsequent prospective large cohort studies. Second, as a single-center study, we only included patients from Xinjiang. Patients from other regions or countries should be included for further validation. Third, no validation cohort was derived for validation the predictive efficacy of the model. Fourthly, based on the AASM scoring manual recommended definition, changes in flow should be associated with a 3% oxygen desaturation or a cortical arousal, although an alternative definition that requires association with a 4% oxygen desaturation without consideration of cortical arousals is accepted [21]. It is important to emphasize that arousals are associated with sympathetic surges leading to atherosclerosis [7, 8, 43]. Thus, in the current study, we did not account for hypopnoeas with arousals might underestimated the severity of OSAS and the effects of OSAS in the pathogenesis of atherosclerosis. Finally, an external validation is still needed for this nomogram.

## 5. Conclusions

In conclusion, this observational study showed that multiple variables of clinical characteristics (hypertension, diabetes mellitus, impaired glucose tolerance, NAFLD, and BMI) and biochemical parameters (HDL-C, LDL-C, NLR, and PLR) were potential predictors for moderate to severe OSAS in patients with CCS. A risk nomogram constructed on the basis of these variables in patients with CCS showed satisfying discriminative ability to predict OSAS comorbidity. Although further evaluation in large-scale studies is needed, results of the study support to use this nomogram for the identification of CCS patients at high-risk for OSAS, which may be helpful to optimize the clinical management of patients with CAD.

## Figures and Tables

**Table 1:** Baseline characteristics of included patients with CCS according to the presence of OSAS.

	CCS patients with no or mild OSAS (*n* = 236)	CCS patients with moderate to severe OSAS (*n* = 291)	*t*/*Z*/*χ*^2^	*P*
Male (%)	160 (67.8)	214 (73.5)	2.086	0.149
Age (years)	57.17 ± 11.06	56.56 ± 10.46	0.640	0.522
Hypertension (%)	173 (73.3)	249 (85.6)	12.281	<0.001
Diabetes mellitus (%)	58 (24.6)	106 (36.4)	8.537	0.003
Impaired glucose tolerance (%)	16 (6.8)	52 (17.9)	14.261	<0.001
Current smoking (%)	102 (43.2)	133 (45.7)	0.325	0.568
Duration of smoking (years)	20.00 (15.00, 30.00)	20.00 (13.00, 30.00)	0.713	0.476
Current smoking cigarettes per day	20.00 (10.00, 20.00)	20.00 (5.00, 20.00)	1.678	0.093
Drinking (%)			3.453	0.178
Never drinking	141 (59.7)	162 (55.7)		
Former drinking	73 (30.9)	110 (37.8)		
Current drinking	22 (9.3)	19 (6.5)		
Family history of CAD (%)	36 (15.3)	42 (14.4)	0.070	0.792
Nonalcoholic fatty liver disease (%)	110 (46.6)	196 (67.4)	23.029	<0.001
Creatinine (mmol/L)	79.06 ± 18.24	79.31 ± 22.75	0.138	0.890
Uric acid (mmol/L)	334.95 ± 81.94	353.14 ± 76.71	2.625	0.009
Serum glucose (mmol/L)	5.97 ± 2.24	6.26 ± 2.61	1.386	0.166
HbA1c %	5.98 ± 0.89	6.22 ± 1.04	2.898	0.004
HDL-C (mmol/L)	1.27 ± 0.35	1.21 ± 0.25	2.118	0.035
LDL-C (mmol/L)	2.05 ± 0.74	2.43 ± 0.78	5.749	<0.001
VLDL -C (mmol/L)	0.67 (0.45, 0.93)	0.62 (0.46, 0.87)	0.981	0.326
TC (mmol/L)	4.04 ± 1.07	4.26 ± 0.96	2.500	0.013
TG (mmol/L)	1.57 (1.13, 2.25)	1.64 (1.18, 2.34)	1.140	0.254
ApoA1 (g/L)	1.24 ± 0.23	1.20 ± 0.18	1.909	0.057
ApoB (g/L)	0.93 ± 0.24	0.90 ± 0.24	1.494	0.136
Lp (a) (g/L)	148.58 (65.58, 301.88)	126.23 (56.90, 270.22)	1.259	0.208
Fibrinogen (g/L)	3.10 ± 0.71	3.18 ± 1.02	0.984	0.326
White blood cell count (10^9^/L)	6.72 ± 1.83	6.86 ± 1.78	0.936	0.350
NLR	1.81 (1.46, 2.39)	2.05 (1.57, 2.61)	3.047	0.002
PLR	82.27 (17.29, 124.17)	99.06 (59.64, 132.12)	2.532	0.011
Hs-CRP (mg/L)	1.04 (0.59, 2.52)	1.00 (0.57, 3.10)	0.136	0.892
NT-pro BNP (pg/ml)	79.25 (32.10, 110.10)	81.78 (33.70, 145.90)	0.826	0.409
LVEF %	63.04 ± 5.47	63.35 ± 5.92	0.586	0.558
LVEDD (mm)	49.94 ± 4.04	49.93 ± 4.20	0.036	0.972
BMI (kg/m^2^)	27.60 ± 3.89	29.74 ± 4.48	5.872	<0.001
Neck circumference (cm)	39.72 ± 4.12	41.92 ± 4.70	5.655	<0.001
SDNN	126.00 (108.00, 149.00)	121.00 (98.00, 150.00)	1.918	0.055
SDANN	102.50 (88.00, 123.75)	98.00 (80.00, 120.00)	2.236	0.035
PNN50	8.00 (4.00, 15.00)	9.00 (4.00, 17.00)	1.447	0.148
RMSSD	71.00 (36.00, 138.50)	80.00 (40.00, 145.00)	1.062	0.288
Average heart rate (beats/min)	72.17 ± 8.02	73.70 ± 9.53	1.977	0.049

OSAS: obstructive sleep apnea syndrome; CSC: chronic coronary syndromes; CAD: coronary artery disease; TC: total cholesterol; TG: triglyceride; HDL-C: high-density lipoprotein cholesterol; LDL-C: low-density lipoprotein-cholesterol; VLDL-C: very low-density lipoprotein cholesterol; Apo-AI: apolipoprotein A1; Apo-B: apolipoprotein B; Lp (a): lipoprotein (a); LVEF: left ventricular ejection fraction (%); LVEDD: left ventricular end diastolic diameter; BMI: body mass index; hs-CRP: high-sensitivity C-reactive protein; NLR: neutrophil to lymphocyte ratio; PLR: platelet-to-lymphocyte ratio; N-terminal pro brain natriuretic peptide (NT-proBNP); SDNN: standard deviation of all normal sinus RR intervals; RMSSD: root mean square successive difference; SDANN: standard deviation average of NN intervals; PNN50: percentage of the number of times that the difference between adjacent normal RR intervals >50 ms over the total number of NN intervals.

**Table 2:** Parameters from the sleep monitoring study in CCS patients according to the presence of OSAS.

	CCS patients with no or mild OSA (*n* = 236)	CCS patients with moderate to severe OSA (*n* = 291)	*t*/*Z*/*χ*^2^	*P*
Obstructive respiratory disturbance index	6.00 (3.80, 8.30)	19.80 (13.20, 31.80)	17.231	<0.001
Time ratio of SpO2<85% (min)	1.00 (0.00, 9.00)	10.00 (2.00, 30.18)	8.988	<0.001
Average blood oxygen saturation	91.97 ± 2.70	89.42 ± 4.23	8.380	<0.001
Lowest blood oxygen saturation	82.22 ± 7.30	74.56 ± 9.39	10.535	<0.001
Lowest heart rate during sleep (beats/min)	56.67 ± 7.54	59.06 ± 9.33	3.260	0.001
Highest heart rate during sleep (beats/min)	78.52 ± 10.17	79.77 ± 13.47	1.181	0.238
Mean heart rate (beats/min)	65.25 ± 7.55	67.98 ± 9.85	3.599	<0.001

**Table 3:** Potential predictors for moderate to severe OSAS in patients with CCS: results of the logistic regression analyses.

	B	SE	Wald	*P*	OR	95% CI	
						Lower limit	Upper limit
Hypertension (%)	0.623	0.257	5.862	0.015	1.865	1.126	3.089
Diabetes mellitus (%)	0.719	0.225	10.227	0.001	2.052	1.321	3.187
Impaired glucose tolerance (%)	1.398	0.333	17.648	<0.001	4.049	2.108	7.774
Nonalcoholic fatty liver disease (%)	0.539	0.207	6.792	0.009	1.715	1.143	2.573
HDL-C (mmol/L)	-0.996	0.397	6.300	0.012	0.369	0.170	0.804
LDL-C (mmol/L)	0.799	0.145	30.242	<0.001	2.224	1.673	2.957
NLR	0.246	0.094	6.782	0.009	1.279	1.063	1.538
PLR	0.006	0.002	9.912	0.002	1.006	1.002	1.009
BMI (kg/m^2^)	0.091	0.026	12.175	<0.001	1.095	1.041	1.152

## Data Availability

The datasets used and/or analyzed during this study are available from the corresponding author on reasonable request. Requests to access these datasets should be directed to Jun Wang, junwang0607@163.com.
