# Corrigendum to “Novel Insights into the Predictors of Obstructive Sleep Apnea Syndrome in Patients with Chronic Coronary Syndrome: Development of a Predicting Model”

**DOI:** 10.1155/2022/9821980

**Published:** 2022-10-27

**Authors:** Yanan Xu, Zongwei Ye, Benfang Wang, Long Tang, Jun Sun, Xuedong Chen, Yi Yang, Jun Wang

**Affiliations:** ^1^Pulmonary and Critical Care Medicine, People's Hospital of Xuancheng City, Anhui, China; ^2^Suzhou Ninth Hospital Affiliated to Soochow University, Jiangsu, China; ^3^Department of Cardiology, The First Affiliated Hospital of Bengbu Medical College, Bengbu, Anhui, China; ^4^Department of Cardiology, People's Hospital of Xuancheng City, Anhui, China; ^5^Xinjiang Medical University, Ürümqi 830011, China; ^6^Department of Cardiology Fourth Ward, The Xinjiang Medical University Affiliated Hospital of Traditional Chinese Medicine, Ürümqi 830011, China

In the article titled “Novel Insights into the Predictors of Obstructive Sleep Apnea Syndrome in Patients with Chronic Coronary Syndrome: Development of a Predicting Model” [1], several writing errors were made when drafting the article where “male” replaced the word “hypertension.” Therefore, the following corrections have been made to the article:

- In Section 3.2, “male” has been corrected to “hypertension”
- In Table 3, “male” has been corrected to “hypertension”
- In Section 3.3, “male sex” has been corrected to “hypertension”
- In the first paragraph of Discussion, “male” has been corrected to “hypertension”
- In Conclusions, “male” has been corrected to “hypertension”
